# Precision subclassification of type 2 diabetes: a systematic review

**DOI:** 10.1038/s43856-023-00360-3

**Published:** 2023-10-05

**Authors:** Shivani Misra, Robert Wagner, Bige Ozkan, Martin Schön, Magdalena Sevilla-Gonzalez, Katsiaryna Prystupa, Caroline C. Wang, Raymond J. Kreienkamp, Sara J. Cromer, Mary R. Rooney, Daisy Duan, Anne Cathrine Baun Thuesen, Amelia S. Wallace, Aaron Leong, Aaron J. Deutsch, Mette K. Andersen, Liana K. Billings, Robert H. Eckel, Wayne Huey-Herng Sheu, Torben Hansen, Norbert Stefan, Mark O. Goodarzi, Debashree Ray, Elizabeth Selvin, Jose C. Florez, ADA/EASD PMDI, Deirdre K. Tobias, Jordi Merino, Abrar Ahmad, Catherine Aiken, Jamie L. Benham, Dhanasekaran Bodhini, Amy L. Clark, Kevin Colclough, Rosa Corcoy, Sara J. Cromer, Jamie L. Felton, Ellen C. Francis, Pieter Gillard, Véronique Gingras, Romy Gaillard, Eram Haider, Alice Hughes, Jennifer M. Ikle, Laura M. Jacobsen, Anna R. Kahkoska, Jarno L. T. Kettunen, Raymond J. Kreienkamp, Lee-Ling Lim, Jonna M. E. Männistö, Robert Massey, Niamh-Maire Mclennan, Rachel G. Miller, Mario Luca Morieri, Jasper Most, Rochelle N. Naylor, Bige Ozkan, Kashyap Amratlal Patel, Scott J. Pilla, Sridaran Raghaven, Martin Schön, Zhila Semnani-Azad, Magdalena Sevilla-Gonzalez, Pernille Svalastoga, Wubet Worku Takele, Claudia Ha-ting Tam, Anne Cathrine B. Thuesen, Mustafa Tosur, Caroline C. Wang, Jessie J. Wong, Jennifer M. Yamamoto, Katherine Young, Chloé Amouyal, Maxine P. Bonham, Mingling Chen, Feifei Cheng, Tinashe Chikowore, Sian C. Chivers, Christoffer Clemmensen, Dana Dabelea, Adem Y. Dawed, Aaron J. Deutsch, Laura T. Dickens, Linda A. DiMeglio, Monika Dudenhöffer-Pfeifer, Carmella Evans-Molina, María Mercè Fernández-Balsells, Hugo Fitipaldi, Stephanie L. Fitzpatrick, Stephen E. Gitelman, Mark O. Goodarzi, Jessica A. Grieger, Marta Guasch-Ferré, Nahal Habibi, Chuiguo Huang, Arianna Harris-Kawano, Heba M. Ismail, Benjamin Hoag, Randi K. Johnson, Angus G. Jones, Robert W. Koivula, Aaron Leong, Gloria K. W. Leung, Ingrid M. Libman, Kai Liu, S. Alice Long, William L. Lowe, Robert W. Morton, Ayesha A. Motala, Suna Onengut-Gumuscu, James S. Pankow, Maleesa Pathirana, Sofia Pazmino, Dianna Perez, John R. Petrie, Camille E. Powe, Alejandra Quinteros, Rashmi Jain, Mathias Ried-Larsen, Zeb Saeed, Vanessa Santhakumar, Sarah Kanbour, Sudipa Sarkar, Gabriela S. F. Monaco, Denise M. Scholtens, Wayne Huey-Herng Sheu, Cate Speake, Maggie A. Stanislawski, Nele Steenackers, Andrea K. Steck, Norbert Stefan, Julie Støy, Rachael Taylor, Sok Cin Tye, Gebresilasea Gendisha Ukke, Marzhan Urazbayeva, Bart Van der Schueren, Camille Vatier, John M. Wentworth, Wesley Hannah, Sara L. White, Gechang Yu, Yingchai Zhang, Shao J. Zhou, Jacques Beltrand, Michel Polak, Ingvild Aukrust, Elisa de Franco, Sarah E. Flanagan, Kristin A. Maloney, Andrew McGovern, Janne Molnes, Mariam Nakabuye, Pål Rasmus Njølstad, Hugo Pomares-Millan, Michele Provenzano, Cécile Saint-Martin, Cuilin Zhang, Yeyi Zhu, Sungyoung Auh, Russell de Souza, Andrea J. Fawcett, Chandra Gruber, Eskedar Getie Mekonnen, Emily Mixter, Diana Sherifali, Robert H. Eckel, John J. Nolan, Louis H. Philipson, Rebecca J. Brown, Liana K. Billings, Kristen Boyle, Tina Costacou, John M. Dennis, Jose C. Florez, Anna L. Gloyn, Maria F. Gomez, Peter A. Gottlieb, Siri Atma W. Greeley, Kurt Griffin, Andrew T. Hattersley, Irl B. Hirsch, Marie-France Hivert, Korey K. Hood, Jami L. Josefson, Soo Heon Kwak, Lori M. Laffel, Siew S. Lim, Ruth J. F. Loos, Ronald C. W. Ma, Chantal Mathieu, Nestoras Mathioudakis, James B. Meigs, Shivani Misra, Viswanathan Mohan, Rinki Murphy, Richard Oram, Katharine R. Owen, Susan E. Ozanne, Ewan R. Pearson, Wei Perng, Toni I. Pollin, Rodica Pop-Busui, Richard E. Pratley, Leanne M. Redman, Maria J. Redondo, Rebecca M. Reynolds, Robert K. Semple, Jennifer L. Sherr, Emily K. Sims, Arianne Sweeting, Tiinamaija Tuomi, Miriam S. Udler, Kimberly K. Vesco, Tina Vilsbøll, Stephen S. Rich, Paul W. Franks, James B. Meigs, Miriam S. Udler

**Affiliations:** 1 Department of Metabolism, Digestion and Reproduction, Imperial College London, London, UK; 2 Department of Diabetes and Endocrinology, Imperial College Healthcare NHS Trust, London, UK; 3 Department of Endocrinology and Diabetology, University Hospital Düsseldorf, Heinrich Heine University Düsseldorf, Moorenstr. 5, 40225 Düsseldorf, Germany; 4 Institute for Clinical Diabetology, German Diabetes Center, Leibniz Center for Diabetes Research at Heinrich Heine University Düsseldorf, Auf’m Hennekamp 65, 40225 Düsseldorf, Germany; 5 German Center for Diabetes Research (DZD), Ingolstädter Landstraße 1, 85764 Neuherberg, Germany; 6 Welch Center for Prevention, Epidemiology, and Clinical Research, Johns Hopkins Bloomberg School of Public Health, Baltimore, MD USA; 7 Ciccarone Center for the Prevention of Cardiovascular Disease, Johns Hopkins School of Medicine, Baltimore, MD USA; 8 Institute of Experimental Endocrinology, Biomedical Research Center, Slovak Academy of Sciences, Bratislava, Slovakia; 9 Clinical and Translational Epidemiology Unit, Massachusetts General Hospital, Boston, MA USA; 10 Programs in Metabolism and Medical & Population Genetics, The Broad Institute of MIT and Harvard, Cambridge, MA USA; 11 Department of Medicine, Harvard Medical School, Boston, MA USA; 12 Diabetes Unit, Division of Endocrinology, Massachusetts General Hospital, Boston, MA USA; 13 Center for Genomic Medicine, Massachusetts General Hospital, Boston, MA USA; 14 Department of Pediatrics, Division of Endocrinology, Boston Children’s Hospital, Boston, MA USA; 15 Department of Epidemiology, Johns Hopkins Bloomberg School of Public Health, Baltimore, MD USA; 16 Division of Endocrinology, Diabetes and Metabolism, Johns Hopkins University School of Medicine, Baltimore, MD USA; 17 Novo Nordisk Foundation Center for Basic Metabolic Research, Faculty of Health and Medical Sciences, University of Copenhagen, Copenhagen, Denmark; 18 Division of General Internal Medicine, Massachusetts General Hospital, 100 Cambridge St 16th Floor, Boston, MA USA; 19 Division of Endocrinology, Diabetes and Metabolism, NorthShore University Health System, Skokie, IL USA; 20 Department of Medicine, Pritzker School of Medicine, University of Chicago, Chicago, IL USA; 21 Division of Endocrinology, Metabolism and Diabetes, University of Colorado School of Medicine, Aurora, CO USA; 22 Institute of Molecular and Genomic Medicine, National Health Research Institute, Miaoli County, Taiwan, ROC; 23 Division of Endocrinology and Metabolism, Taichung Veterans General Hospital, Taichung, Taiwan, ROC; 24 Division of Endocrinology and Metabolism, Taipei Veterans General Hospital, Taipei, Taiwan, ROC; 25 University Hospital of Tübingen, Tübingen, Germany; 26 Institute of Diabetes Research and Metabolic Diseases (IDM), Helmholtz Center Munich, Neuherberg, Germany; 27 Division of Endocrinology, Diabetes and Metabolism, Department of Medicine, Cedars-Sinai Medical Center, Los Angeles, CA USA; 28 Department of Biostatistics, Johns Hopkins Bloomberg School of Public Health, Baltimore, MD USA; 29 Division of Preventative Medicine, Department of Medicine, Brigham and Women’s Hospital and Harvard Medical School, Boston, MA USA; 30 Department of Nutrition, Harvard T.H. Chan School of Public Health, Boston, MA USA; 31 Diabetes Unit, Endocrine Division, Massachusetts General Hospital, Boston, MA USA; 32 Department of Clinical Sciences, Lund University Diabetes Centre, Lund University, Malmö, Sweden; 33 Department of Obstetrics and Gynaecology, the Rosie Hospital, Cambridge, UK; 34 NIHR Cambridge Biomedical Research Centre, University of Cambridge, Cambridge, UK; 35 Departments of Medicine and Community Health Sciences, Cumming School of Medicine, University of Calgary, Calgary, AB Canada; 36 Department of Molecular Genetics, Madras Diabetes Research Foundation, Chennai, India; 37 Division of Pediatric Endocrinology, Department of Pediatrics, Saint Louis University School of Medicine, SSM Health Cardinal Glennon Children’s Hospital, St. Louis, MO USA; 38 Department of Clinical and Biomedical Sciences, University of Exeter Medical School, Exeter, Devon UK; 39 CIBER-BBN, ISCIII, Madrid, Spain; 40 Institut d’Investigació Biomèdica Sant Pau (IIB SANT PAU), Barcelona, Spain; 41 Departament de Medicina, Universitat Autònoma de Barcelona, Bellaterra, Spain; 42 Programs in Metabolism and Medical & Population Genetics, Broad Institute, Cambridge, MA USA; 43 Department of Pediatrics, Indiana University School of Medicine, Indianapolis, IN USA; 44 Herman B Wells Center for Pediatric Research, Indiana University School of Medicine, Indianapolis, IN USA; 45 Center for Diabetes and Metabolic Diseases, Indiana University School of Medicine, Indianapolis, IN USA; 46 Department of Biostatistics and Epidemiology, Rutgers School of Public Health, Piscataway, NJ USA; 47 University Hospital Leuven, Leuven, Belgium; 48 Department of Nutrition, Université de Montréal, Montreal, QC Canada; 49 Research Center, Sainte-Justine University Hospital Center, Montreal, QC Canada; 50 Department of Pediatrics, Erasmus Medical Center, Rotterdam, The Netherlands; 51 Division of Population Health & Genomics, School of Medicine, University of Dundee, Dundee, UK; 52 Department of Pediatrics, Stanford School of Medicine, Stanford University, Stanford, CA USA; 53 Stanford Diabetes Research Center, Stanford School of Medicine, Stanford University, Stanford, CA USA; 54 University of Florida, Gainesville, FL USA; 55 Department of Nutrition, University of North Carolina at Chapel Hill, Chapel Hill, NC USA; 56 Helsinki University Hospital, Abdominal Centre/Endocrinology, Helsinki, Finland; 57 Folkhalsan Research Center, Helsinki, Finland; 58 Institute for Molecular Medicine Finland FIMM, University of Helsinki, Helsinki, Finland; 59 Department of Medicine, Faculty of Medicine, University of Malaya, Kuala Lumpur, Malaysia; 60 Asia Diabetes Foundation, Hong Kong SAR, China; 61 Department of Medicine & Therapeutics, Chinese University of Hong Kong, Hong Kong SAR, China; 62 Departments of Pediatrics and Clinical Genetics, Kuopio University Hospital, Kuopio, Finland; 63 Department of Medicine, University of Eastern Finland, Kuopio, Finland; 64 Centre for Cardiovascular Science, Queen’s Medical Research Institute, University of Edinburgh, Edinburgh, UK; 65 Department of Epidemiology, University of Pittsburgh, Pittsburgh, PA USA; 66 Metabolic Disease Unit, University Hospital of Padova, Padova, Italy; 67 Department of Medicine, University of Padova, Padova, Italy; 68 Department of Orthopedics, Zuyderland Medical Center, Sittard-Geleen, The Netherlands; 69 Departments of Pediatrics and Medicine, University of Chicago, Chicago, IL USA; 70 Ciccarone Center for the Prevention of Cardiovascular Disease, Johns Hopkins School of Medicine, Baltimore, MD USA; 71 Department of Medicine, Johns Hopkins University, Baltimore, MD USA; 72 Department of Health Policy and Management, Johns Hopkins University Bloomberg School of Public Health, Baltimore, MD USA; 73 Section of Academic Primary Care, US Department of Veterans Affairs Eastern Colorado Health Care System, Aurora, CO USA; 74 Department of Medicine, University of Colorado School of Medicine, Aurora, CO USA; 75 Institute of Experimental Endocrinology, Biomedical Research Center, Slovak Academy of Sciences, Bratislava, Slovakia; 76 Mohn Center for Diabetes Precision Medicine, Department of Clinical Science, University of Bergen, Bergen, Norway; 77 Children and Youth Clinic, Haukeland University Hospital, Bergen, Norway; 78 Eastern Health Clinical School, Monash University, Melbourne, VIC Australia; 79 Laboratory for Molecular Epidemiology in Diabetes, Li Ka Shing Institute of Health Sciences, The Chinese University of Hong Kong, Hong Kong, China; 80 Hong Kong Institute of Diabetes and Obesity, The Chinese University of Hong Kong, Hong Kong, China; 81 Department of Pediatrics, Baylor College of Medicine, Houston, TX USA; 82 Division of Pediatric Diabetes and Endocrinology, Texas Children’s Hospital, Houston, TX USA; 83 Children’s Nutrition Research Center, USDA/ARS, Houston, TX USA; 84 Stanford University School of Medicine, Stanford, CA USA; 85 Internal Medicine, University of Manitoba, Winnipeg, MB Canada; 86 Department of Diabetology, APHP, Paris, France; 87 Sorbonne Université, INSERM, NutriOmic team, Paris, France; 88 Department of Nutrition, Dietetics and Food, Monash University, Melbourne, VIC Australia; 89 Monash Centre for Health Research and Implementation, Monash University, Clayton, VIC Australia; 90 Health Management Center, The Second Affiliated Hospital of Chongqing Medical University, Chongqing Medical University, Chongqing, China; 91 MRC/Wits Developmental Pathways for Health Research Unit, Department of Paediatrics, Faculty of Health Sciences, University of the Witwatersrand, Johannesburg, South Africa; 92 Channing Division of Network Medicine, Brigham and Women’s Hospital, Boston, MA USA; 93 Sydney Brenner Institute for Molecular Bioscience, Faculty of Health Sciences, University of the Witwatersrand, Johannesburg, South Africa; 94 Department of Women and Children’s health, King’s College London, London, UK; 95 Lifecourse Epidemiology of Adiposity and Diabetes (LEAD) Center, University of Colorado Anschutz Medical Campus, Aurora, CO USA; 96 Section of Adult and Pediatric Endocrinology, Diabetes and Metabolism, Kovler Diabetes Center, University of Chicago, Chicago, IL USA; 97 Department of Pediatrics, Riley Hospital for Children, Indiana University School of Medicine, Indianapolis, IN USA; 98 Richard L. Roudebush VAMC, Indianapolis, IN USA; 99 Biomedical Research Institute Girona, IdIBGi, Girona, Spain; 100 Diabetes, Endocrinology and Nutrition Unit Girona, University Hospital Dr Josep Trueta, Girona, Spain; 101 Institute of Health System Science, Feinstein Institutes for Medical Research, Northwell Health, Manhasset, NY USA; 102 University of California at San Francisco, Department of Pediatrics, Diabetes Center, San Francisco, CA USA; 103 Division of Endocrinology, Diabetes and Metabolism, Cedars-Sinai Medical Center, Los Angeles, CA USA; 104 Department of Medicine, Cedars-Sinai Medical Center, Los Angeles, CA USA; 105 Adelaide Medical School, Faculty of Health and Medical Sciences, The University of Adelaide, Adelaide, SA Australia; 106 Robinson Research Institute, The University of Adelaide, Adelaide, SA Australia; 107 Department of Public Health and Novo Nordisk Foundation Center for Basic Metabolic Research, Faculty of Health and Medical Sciences, University of Copenhagen, 1014 Copenhagen, Denmark; 108 Division of Endocrinology and Diabetes, Department of Pediatrics, Sanford Children’s Hospital, Sioux Falls, SD USA; 109 University of South Dakota, School of Medicine, E Clark St, Vermillion, SD USA; 110 Department of Biomedical Informatics, University of Colorado Anschutz Medical Campus, Aurora, CO USA; 111 Department of Epidemiology, Colorado School of Public Health, Aurora, CO USA; 112 Royal Devon University Healthcare NHS Foundation Trust, Exeter, UK; 113 Oxford Centre for Diabetes, Endocrinology and Metabolism, University of Oxford, Oxford, UK; 114 Division of General Internal Medicine, Massachusetts General Hospital, Boston, MA USA; 115 UPMC Children’s Hospital of Pittsburgh, Pittsburgh, PA USA; 116 Center for Translational Immunology, Benaroya Research Institute, Seattle, WA USA; 117 Department of Medicine, Northwestern University Feinberg School of Medicine, Chicago, IL USA; 118 Department of Pathology & Molecular Medicine, McMaster University, Hamilton, ON Canada; 119 Population Health Research Institute, Hamilton, ON Canada; 120 Department of Translational Medicine, Medical Science, Novo Nordisk Foundation, Tuborg Havnevej 19, 2900 Hellerup, Denmark; 121 Department of Diabetes and Endocrinology, Nelson R Mandela School of Medicine, University of KwaZulu-Natal, Durban, South Africa; 122 Center for Public Health Genomics, Department of Public Health Sciences, University of Virginia, Charlottesville, VA USA; 123 Division of Epidemiology and Community Health, School of Public Health, University of Minnesota, Minneapolis, MN USA; 124 Department of Chronic Diseases and Metabolism, Clinical and Experimental Endocrinology, KU Leuven, Leuven, Belgium; 125 School of Health and Wellbeing, College of Medical, Veterinary and Life Sciences, University of Glasgow, Glasgow, UK; 126 Department of Obstetrics, Gynecology, and Reproductive Biology, Massachusetts General Hospital and Harvard Medical School, Boston, MA USA; 127 Sanford Children’s Specialty Clinic, Sioux Falls, SD USA; 128 Department of Pediatrics, Sanford School of Medicine, University of South Dakota, Sioux Falls, SD USA; 129 Centre for Physical Activity Research, Rigshospitalet, Copenhagen, Denmark; 130 Institute for Sports and Clinical Biomechanics, University of Southern Denmark, Odense, Denmark; 131 Department of Medicine, Division of Endocrinology, Diabetes and Metabolism, Indiana University School of Medicine, Indianapolis, IN USA; 132 AMAN Hospital, Doha, Qatar; 133 Department of Preventive Medicine, Division of Biostatistics, Northwestern University Feinberg School of Medicine, Chicago, IL USA; 134 Institute of Molecular and Genomic Medicine, National Health Research Institutes, Taipei City, Taiwan, ROC; 135 Divsion of Endocrinology and Metabolism, Taichung Veterans General Hospital, Taichung, Taiwan, ROC; 136 Center for Interventional Immunology, Benaroya Research Institute, Seattle, WA USA; 137 Barbara Davis Center for Diabetes, University of Colorado Anschutz Medical Campus, Aurora, CO USA; 138 Institute of Diabetes Research and Metabolic Diseases (IDM), Helmholtz Center Munich, Neuherberg, Germany; 139 Steno Diabetes Center Aarhus, Aarhus University Hospital, Aarhus, Denmark; 140 University of Newcastle, Newcastle upon Tyne, UK; 141 Section of Genetics and Epidemiology, Joslin Diabetes Center, Harvard Medical School, Boston, MA USA; 142 Department of Clinical Pharmacy and Pharmacology, University Medical Center Groningen, Groningen, The Netherlands; 143 Department of Gastroenterology, Baylor College of Medicine, Houston, TX USA; 144 Department of Endocrinology, University Hospitals Leuven, Leuven, Belgium; 145 Sorbonne University, Inserm U938, Saint-Antoine Research Centre, Institute of Cardiometabolism and Nutrition, Paris, 75012 France; 146 Department of Endocrinology, Diabetology and Reproductive Endocrinology, Assistance Publique-Hôpitaux de Paris, Saint-Antoine University Hospital, National Reference Center for Rare Diseases of Insulin Secretion and Insulin Sensitivity (PRISIS), Paris, France; 147 Department of Diabetes and Endocrinology, Royal Melbourne Hospital, Parkville, VIC Australia; 148 Walter and Eliza Hall Institute, Parkville, VIC Australia; 149 Department of Medicine, University of Melbourne, Parkville, VIC Australia; 150 Deakin University, Melbourne, VIC Australia; 151 Department of Epidemiology, Madras Diabetes Research Foundation, Chennai, India; 152 Department of Diabetes and Endocrinology, Guy’s and St Thomas’ Hospitals NHS Foundation Trust, London, UK; 153 School of Agriculture, Food and Wine, University of Adelaide, Adelaide, Australia; 154 Institut Cochin, Inserm U, 10116 Paris, France; 155 Pediatric Endocrinology and Diabetes, Hopital Necker Enfants Malades, APHP Centre, Université de Paris, Paris, France; 156 Department of Medical Genetics, Haukeland University Hospital, Bergen, Norway; 157 Department of Medicine, University of Maryland School of Medicine, Baltimore, MD USA; 158 Department of Epidemiology, Geisel School of Medicine at Dartmouth, Hanover, NH USA; 159 Nephrology, Dialysis and Renal Transplant Unit, IRCCS—Azienda Ospedaliero-Universitaria di Bologna, Alma Mater Studiorum University of Bologna, Bologna, Italy; 160 Department of Medical Genetics, AP-HP Pitié-Salpêtrière Hospital, Sorbonne University, Paris, France; 161 Global Center for Asian Women’s Health, Yong Loo Lin School of Medicine, National University of Singapore, Singapore, Singapore; 162 Department of Obstetrics and Gynecology, Yong Loo Lin School of Medicine, National University of Singapore, Singapore, Singapore; 163 Division of Research, Kaiser Permanente Northern California, Oakland, CA USA; 164 Department of Epidemiology and Biostatistics, University of California San Francisco, California, USA; 165 National Institute of Diabetes and Digestive and Kidney Diseases, National Institutes of Health, Bethesda, MD USA; 166 Department of Health Research Methods, Evidence, and Impact, Faculty of Health Sciences, McMaster University, Hamilton, ON Canada; 167 Ann & Robert H. Lurie Children’s Hospital of Chicago, Department of Pediatrics, Northwestern University Feinberg School of Medicine, Chicago, IL USA; 168 Department of Clinical and Organizational Development, Children’s Memorial Hospital, Chicago, IL USA; 169 American Diabetes Association, Arlington, VA USA; 170 College of Medicine and Health Sciences, University of Gondar, Gondar, Ethiopia; 171 Faculty of Medicine and Health Sciences, Global Health Institute, University of Antwerp, 2160 Antwerp, Belgium; 172 Department of Medicine and Kovler Diabetes Center, University of Chicago, Chicago, IL USA; 173 School of Nursing, Faculty of Health Sciences, McMaster University, Hamilton, ON Canada; 174 Division of Endocrinology, Metabolism, Diabetes, University of Colorado, Boulder, CO USA; 175 Department of Clinical Medicine, School of Medicine, Trinity College Dublin, Dublin, Ireland; 176 Department of Endocrinology, Wexford General Hospital, Wexford, Ireland; 177 Division of Endocrinology, NorthShore University HealthSystem, Skokie, IL USA; 178 Department of Medicine, Prtizker School of Medicine, University of Chicago, Chicago, IL USA; 179 Department of Genetics, Stanford School of Medicine, Stanford University, Stanford, CA USA; 180 Faculty of Health, Aarhus University, Aarhus, Denmark; 181 Departments of Pediatrics and Medicine and Kovler Diabetes Center, University of Chicago, Chicago, USA; 182 Sanford Research, Sioux Falls, SD USA; 183 University of Washington School of Medicine, Seattle, WA USA; 184 Department of Population Medicine, Harvard Medical School, Harvard Pilgrim Health Care Institute, Boston, MA USA; 185 Department of Medicine, Universite de Sherbrooke, Sherbrooke, QC Canada; 186 Department of Internal Medicine, Seoul National University College of Medicine, Seoul National University Hospital, Seoul, Republic of Korea; 187 Joslin Diabetes Center, Harvard Medical School, Boston, MA USA; 188 Charles Bronfman Institute for Personalized Medicine, Icahn School of Medicine at Mount Sinai, New York, NY USA; 189 Broad Institute, Cambridge, MA USA; 190 Division of Metabolism, Digestion and Reproduction, Imperial College London, London, UK; 191 Department of Diabetes & Endocrinology, Imperial College Healthcare NHS Trust, London, UK; 192 Department of Diabetology, Madras Diabetes Research Foundation & Dr. Mohan’s Diabetes Specialities Centre, Chennai, India; 193 Department of Medicine, Faculty of Medicine and Health Sciences, University of Auckland, Auckland, New Zealand; 194 Auckland Diabetes Centre, Te Whatu Ora Health New Zealand, Auckland, New Zealand; 195 Medical Bariatric Service, Te Whatu Ora Counties, Health New Zealand, Auckland, New Zealand; 196 Oxford NIHR Biomedical Research Centre, University of Oxford, Oxford, UK; 197 Metabolic Research Laboratories and MRC Metabolic Diseases Unit, University of Cambridge, Wellcome-MRC Institute of Metabolic Science, Cambridge, UK; 198 Department of Epidemiology & Public Health, University of Maryland School of Medicine, Baltimore, MD USA; 199 Department of Internal Medicine, Division of Metabolism, Endocrinology and Diabetes, University of Michigan, Ann Arbor, MI USA; 200 AdventHealth Translational Research Institute, Orlando, FL USA; 201 Pennington Biomedical Research Center, Baton Rouge, LA USA; 202 MRC Human Genetics Unit, Institute of Genetics and Cancer, University of Edinburgh, Edinburgh, UK; 203 Yale School of Medicine, New Haven, CT USA; 204 Faculty of Medicine and Health, University of Sydney, Sydney, NSW Australia; 205 Department of Endocrinology, Royal Prince Alfred Hospital, Sydney, NSW Australia; 206 Kaiser Permanente Northwest, Kaiser Permanente Center for Health Research, Portland, OR USA; 207 Clinial Research, Steno Diabetes Center Copenhagen, Herlev, Denmark; 208 Department of Clinical Medicine, Faculty of Health and Medical Sciences, University of Copenhagen, Copenhagen, Denmark

**Keywords:** Type 2 diabetes, Body mass index

## Abstract

**Background:**

Heterogeneity in type 2 diabetes presentation and progression suggests that precision medicine interventions could improve clinical outcomes. We undertook a systematic review to determine whether strategies to subclassify type 2 diabetes were associated with high quality evidence, reproducible results and improved outcomes for patients.

**Methods:**

We searched PubMed and Embase for publications that used ‘simple subclassification’ approaches using simple categorisation of clinical characteristics, or ‘complex subclassification’ approaches which used machine learning or ‘omics approaches in people with established type 2 diabetes. We excluded other diabetes subtypes and those predicting incident type 2 diabetes. We assessed quality, reproducibility and clinical relevance of extracted full-text articles and qualitatively synthesised a summary of subclassification approaches.

**Results:**

Here we show data from 51 studies that demonstrate many simple stratification approaches, but none have been replicated and many are not associated with meaningful clinical outcomes. Complex stratification was reviewed in 62 studies and produced reproducible subtypes of type 2 diabetes that are associated with outcomes. Both approaches require a higher grade of evidence but support the premise that type 2 diabetes can be subclassified into clinically meaningful subtypes.

**Conclusion:**

Critical next steps toward clinical implementation are to test whether subtypes exist in more diverse ancestries and whether tailoring interventions to subtypes will improve outcomes.

## Plain language summary

In people with type 2 diabetes there may be differences in the way people present, including for example, their symptoms, body weight or how much insulin they make. We looked at recent publications describing research in this area to see whether it is possible to separate people with type 2 diabetes into different subgroups and, if so, whether these groupings were useful for patients. We found that it is possible to group people with type 2 diabetes into different subgroups and being in one subgroup can be more strongly linked to the likelihood of developing complications over others. This might mean that in the future we can treat people in different subgroups differently in ways that improves their treatment and their health but it requires further study.

## Abstract (web summary)

Misra, Wagner et al. systematically review if strategies to subclassify type 2 diabetes (T2D) are associated with high quality evidence and patient outcomes. Cluster-based stratification yields T2D subtypes that associate with outcomes, suggesting subclassification could have future clinical use.

## Introduction

Type 2 diabetes is a global health problem posing substantial burdens on human health^1^. The diagnosis of type 2 diabetes is based on elevated blood glucose coupled with the absence of clinical features indicating alternative subtypes, such as type 1, monogenic, pancreatic or medication-induced diabetes^2^. A diagnosis of type 2 diabetes is generally the default or can be arrived at through exclusion of other types. Traditionally, most type 2 diabetes care guidelines have advocated treatment choice based on cost-effectiveness and side effects of specific medications, which have no relationship to underlying pathophysiology in the individual. More recent guidelines have suggested differential glucose-lowering therapies on the basis of higher body mass index (BMI) (favouring use of glucagon-like peptide analogue, GLP-1) or presence or absence of cardiovascular and/or renal disease and/or heart failure (favouring GLP-1 and/or sodium-glucose co-transporter 2, SGLT-2 inhibitors)^3^.

There is considerable heterogeneity in the clinical characteristics of patients with type 2 diabetes. Clinicians recognise that differences in degree of obesity or body fat distribution, age, dyslipidaemia or presence of metabolic syndrome can influence prognosis in diabetes and can be important considerations in treatment and management^4–6^. There is increasing awareness that type 2 diabetes heterogeneity may reflect differences in the underlying pathophysiology, environmental contributors, and the genetic risk of affected individuals. The mechanisms leading to the development of type 2 diabetes may differ from one individual to another and this could impact treatment and outcome.

Accurate characterisation of the heterogeneity in type 2 diabetes may help individualise care and improve outcomes. This goal has been realised in part for monogenic diabetes, where treatments can be tailored to genetic subtype to deliver precision care achieving better outcomes than standard care^7^. Given the complex pathophysiology and genetics of type 2 diabetes, applying precision medicine approaches is challenging. Critical to this endeavour is a better understanding of specific subtypes.

There are many studies of type 2 diabetes subtypes. The literature reflects diverse approaches based on the presence or absence of one or more simple clinical features or biomarkers and, more recently, sophisticated methods that deploy machine learning (ML) or use omics data. Classification approaches such as clustering methods to categorise this heterogeneity show inter-cluster differences in progression to complications or need for insulin treatment. These approaches consider clinical features at diagnosis^8^ or clinical information combined with genetic data to characterise disease heterogeneity^9,10^. Simpler approaches are more easily implemented across all resource settings, while complex approaches may have greater precision in classifying heterogeneity. The breadth and scope of the evidence in favour of type 2 diabetes subclassification have not to date been thoroughly examined.

The *Precision Medicine in Diabetes Initiative* (PMDI) was established in 2018 by the American Diabetes Association (ADA) in partnership with the European Association for the Study of Diabetes (EASD). The ADA/EASD PMDI includes global thought leaders in precision diabetes medicine who are working to address the burgeoning need for better diabetes prevention and care through precision medicine^11^. This Systematic Review is written with the ADA/EASD PMDI as part of a comprehensive evidence evaluation in support of the 2nd International Consensus Report on Precision Diabetes Medicine^12^.

In this systematic review for the PMDI we aimed to provide a critical assessment of the evidence to date for type 2 diabetes subclassification using (i) simple approaches based on categorisation of clinical features, biomarkers, imaging, or other parameters, and (ii) complex subclassification approaches that use ML incorporating clinical data and/or genomic data. We aimed to identify areas where further research is needed with the goal to improve patient and health system outcomes in type 2 diabetes care.

Our analysis shows that many simple approaches to subclassification have been tried but none have been replicated and most are not associated with meaningful clinical outcomes. However, a more complex stratification, using machine learning applied to clinical variables, yielded reproducible subtypes of type 2 diabetes that are associated with outcomes. Both approaches, however, require a higher grade of evidence but support the premise that type 2 diabetes can be subclassified into clinically meaningful subtypes.

## Methods

This systematic review was written and conducted in accordance with our pre-established protocol (PROSPERO ID CRD42022310539) and reported using the Preferred Reporting Items for Systematic Reviews and Meta-Analyses Statement (PRISMA)^13^. We systematically reviewed papers to address two research questions devised by an expert working group: 1) What are the main subtypes of type 2 diabetes defined using simple clinical criteria and/or routinely available laboratory tests (simple approaches), and 2) What subphenotypes of type 2 diabetes can be reproducibly identified using ML and/or genomics approaches (complex approaches)? Subsequently, we refer to the first question as *simple approaches* and the second question as *complex approaches*. The quality of each paper was reported, and the aggregate of data evaluated using the Grading of Recommendations, Assessment, Development, and Evaluations (GRADE) system^14^.

### Study eligibility criteria

We included English-language original research studies of all design types that analysed populations with prevalent or new-onset type 2 diabetes and attempted in some way to stratify or subgroup patients with type 2 diabetes. We used broad terms to identify stratification studies and all approaches to stratification (the exposure) were included (supplementary table 1). We excluded studies examining risk for the development of type 2 diabetes, use of glycaemic control (e.g. HbA1c strata) alone to stratify, studies of stratification in types of diabetes other than type 2 diabetes, and review articles or case reports.

For simple approaches the exposure was defined as any of the following; a routine blood or urine biomarker that was widely available in most clinic settings; a blood or urine biomarker that might not be routinely available now but could have the potential to become easily accessible; any routinely available imaging modality; any physiological assessment that could be undertaken in an outpatient setting or results from routinely available dynamic tests. The stratification approach was either a cut-off or categorisation based on one or more of the above or if an index, ratio, trend or other analysis was undertaken, it could be calculated without complex mathematics. Finally, all outcomes were accepted for example clinical characterisation of subgroups, association with specific biomarkers and association with complications or mortality.

For complex approaches, the exposure used was defined as any of the inputs for the simple approach outlined above and/or any form of genetic data. However, unlike the simple approach, the stratification approach either deployed ML approaches or used other complex statistical approaches for stratification. All outcomes were accepted, as above, for simple.

### Literature search and selection strategy

PUBMED and EMBASE databases were searched from inception to May 2022 for relevant articles using a strategy devised by expert health sciences librarians (supplementary methods). We undertook independent searches for each systematic review question. From both searches, each abstract and subsequently, full text paper, was screened by two independent team members for eligibility. In addition to the initial exclusion criteria, at the full-text review stage, we further excluded studies where exposures were not clearly defined and/or if the data on outcomes of the stratification were not available in results or supplementary material. We also excluded studies where the *only* stratification modality was a measure of glycaemic control, as this itself provides the diagnosis of type 2 diabetes. In cases of disagreement between two reviewers, a third reviewer made the final decision. The process involved group-based discussions to resolve disagreements to ensure all decisions were made on the same grounds.

### Data extraction

Data were manually extracted from each full-text paper by individual team members and cross-checked by an independent team member at the data synthesis stage. We extracted relevant data on study design (observational or clinical trial), analysis design (cross-sectional or prospective), study population characteristics, stratification method and results (exposure), outcomes, and study quality assessment. For population characteristics, we extracted data on whether the type 2 diabetes population was new-onset or prevalent, the sample size, ethnicity and gender, the duration of diabetes (for cross-sectional analysis) and duration of follow-up (for longitudinal follow-up). For exposures, we extracted the approach to stratification and the number and nature of subgroups identified. For outcomes, we documented the type of outcome studied and the findings according to stratified subgroup.

### Data synthesis

Following full-text data extraction, we undertook a qualitative analysis of exposures (measures used to stratify individuals) for each systematic review question. For simple sub-classification approaches, we extracted the details of stratification criteria in each paper (supplementary methods), then categorised the exposure as blood/urine test, imaging, age). After data extraction, these exposures were further refined into subcategories based on common emerging themes (e.g., use of pancreatic autoantibodies, BMI categories, measures of beta-cell function, use of lipid profiles). For complex approaches, the exposure included both the input clinical and/or genetic data used and the ML approach to analysis (e.g., k-means, hierarchical clustering, latent-class analysis), deployed. In both reviews, outcomes were heterogeneous, so we broadly categorised them where possible. Due to the variability in exposures and outcomes, it was not possible to undertake formal meta-analyses of any outcome. All coding, categorisation and thematic synthesis was undertaken and agreed upon by at least three members of the research team.

### Quality assessment

The GRADE system was used to assess the quality of the studies extracted^13^. At least two members assessed whether study exposures and outcomes were clearly defined, valid and reliable, and whether confounders were appropriately accounted and adjusted for. Disagreements were resolved by discussion between the joint first and senior authors during group discussion. Assessors evaluated study limitations, consistency of results, imprecision, and reporting bias to assign study-specific and overall GRADE certainty ratings as very low, low, moderate and high^15^.

### Reporting summary

Further information on research design is available in the Nature Portfolio Reporting Summary linked to this article.

## Results

### Search and screening for simple and complex systematic review questions

**Fig. 1:**
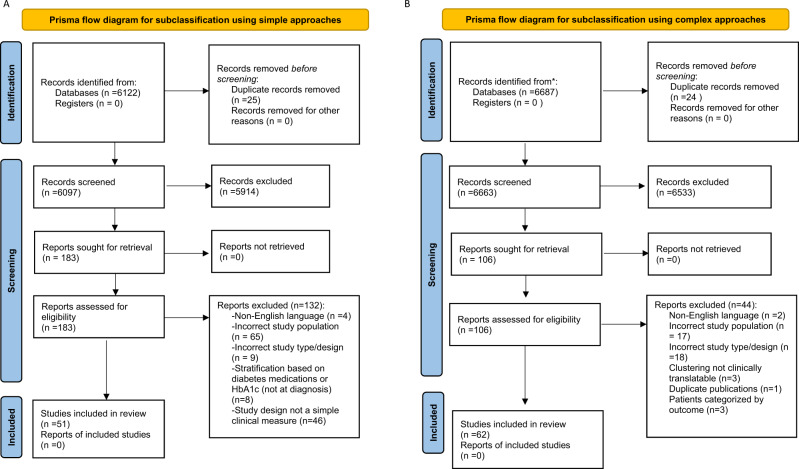
PRISMA systematic review attrition diagram. **A** This shows the flow diagram for simple approaches to subclassification and **B** Complex approaches.

The first question examined simple stratification approaches using clinical variables that may reveal type 2 diabetes heterogeneity. A total of 6097 studies met the inclusion criteria and were screened (Fig. 1A). Of these, 183 studies were included for full text data review, of which 132 studies were subsequently excluded. The most common reasons for exclusion at the full-text review stage were studies conducted in populations without prevalent or incident type 2 diabetes, study designs that used ML approaches or stratification approaches that used HbA1c or diabetes medications. In total, 51 “simple approach” studies underwent full-text data extraction.

The second question aimed to identify papers with complex approaches, mostly ML-based strategies, to identify subgroups of patients with type 2 diabetes (Fig. 1B). A total of 6639 studies were screened, of which 106 were found eligible for full-text review. The most common reasons for exclusion were study populations not comprising participants with type 2 diabetes or classification approaches not using ML. In total, 62 ‘complex’ studies underwent full-text data extraction.

#### Use of simple approaches to subclassify type 2 diabetes

##### Description of extracted studies

The 51 studies using simple type 2 diabetes subclassification approaches incorporated 1,751,350 participants with prevalent or new-onset type 2 diabetes. Among them, 39% (20/51) of studies included participants of white European ancestry, 43% (22/51) incorporated exclusively participants from non-white European ancestries and 17% (9/51) included mixed ancestry groups (Supplementary Data 1). The majority of the studies (78%, 40/51) were conducted in populations with prevalent type 2 diabetes, and 22% (11/51) in new-onset type 2 diabetes. Approximately half the studies had a prospective design (25/51), the remaining half had a cross-sectional (26/51) design. For longitudinal studies, study follow-up periods ranged from <1 year to 22 years.

**Fig. 2:**
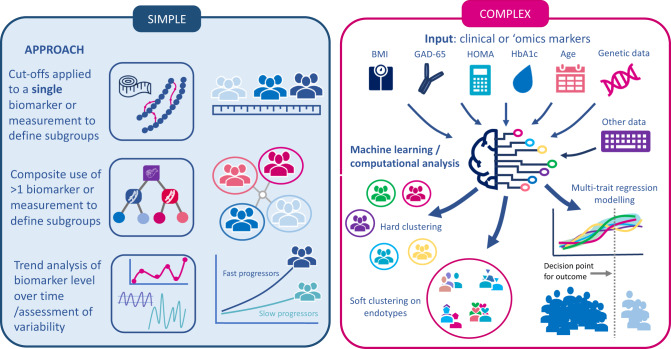
Schematic overview of approaches used to subclassify type 2 diabetes. The figure summarises simple approaches that have been taken to subclassify type 2 diabetes and complex approaches. HbA1c glycated haemoglobin, BMI body mass index, GAD-65 glutamic acid decarboxylase-65 antibodies.

Studies included a wide range of exposures (Fig. 2) based on routine clinical measurements with standard cut-offs or groupings. These included assessment of individual routine clinic-based measurements (e.g., levels of BMI, or biomarker variability over time) or composite stratification incorporating two or more tiers of criteria (e.g. groupings combining one or more biomarkers or anthropometric measurements) including both routine and non-routine but clinically available tests, including oral glucose tolerance tests (OGTT) which, while a glycaemic test, also indirectly measures insulin resistance. The associations of stratified exposure characteristics were investigated with various outcomes: 1) measures of glycaemia, 2) clinical characteristics, 3) measures of diabetes progression such as time-to-insulin treatment or development of microvascular complications and 4) cardiovascular outcomes and/or mortality.

##### Description of categorised subgroups

Simple approaches to classification included use of lipid profiles (*n* = 8), BMI (*n* = 6), pancreatic beta-cell related measures (*n* = 6), pancreatic autoantibodies (*n* = 6), age at diagnosis (*n* = 2), OGTT data (*n* = 4), cardiovascular measures (*n* = 3), other biomarkers in urine or blood and alternative approaches (*n* = 5) (Table 1).

**Table 1:** Summary of published studies using simple approaches to type 2 diabetes classification.

Approach Total n participants % Female Mean age	Race/ethnicity breakdown, %	% Prevalent or new onset T2D, n (%) Prospective or Cross-sectional, *n* (%)	T2D subclassified Groups identified	Outcome category, *n* (%)	Overall quality, *n* (%)	References
Simple: Pancreatic autoantibodies (6 studies) *n* = 8350 42.2% female 54.6 years	87% European/White 11% East Asian (Korean) 2% Black (Nigerian)	Prevalent (*n* = 5, 83%) New onset (*n* = 1, 17%) Longitudinal (*n* = 2, 33%) Cross-sectional (n = 4, 67%)	1) GAD antibody positive vs negative 2) GAD status sub-stratified by age 3) High and low titre GAD positivity	1) Earlier time to insulin treatment (*n* = 2, 33%) 2) Association with variable clinical characteristics (*n* = 3, 50%) 3) Beta-cell function physiological measure (*n* = 1, 17%)	Moderate (*n* = 4, 67%) Low (*n* = 1, 17%) Very low (*n* = 1, 17%)	^20–24,55^
Simple: Beta-cell related measure or index (6 studies) *n* = 17,881 38.1% female 56.9 years	75% East Asian (Chinese, Japanese) 24% European/White, 1% South Asian (Indian)	Prevalent (*n* = 4, 66%) New onset (*n* = 2, 33%) Longitudinal (*n* = 1, 17%) Cross-sectional (*n* = 5, 83%)	1) High versus normal/low fasting insulin/C-peptide level/UCPCR level 2) Insulin sensitive versus resistant	1) Biochemical measures (*n* = 1, 16%) 2) Micro/macrovascular disease (*n* = 3, 50%) 3) Response to medications (*n* = 2, 33%)	Moderate (*n* = 2, 33%) Very low (*n* = 4, 66%)	^56–61^
Simple: Age at diagnosis (2 studies) *n* = 1535 47.6% female Mean age not reported	57% European/White 43% South Asian (Indian)	Prevalent (*n* = 2, 100%) Longitudinal (*n* = 2, 100%)	1) Age 60-75 years vs. >75 years. 2) Age at diagnosis <25 years v. >25 years	1) Total and cardiovascular mortality (*n* = 1, 50%) 2) Severity of retinal disease (*n* = 1, 50%)	Low (*n* = 2, 100%)	^4,6^
Simple: BMI (6 studies) *n* = 74,104 41.6% female 62 years	25% European/White 23% Asian (Chinese, South Asian, Other) 2% non-White 50% not reported	Prevalent (*n* = 6, 100%) Longitudinal (*n* = 4, 67%) Cross-sectional (*n* = 2, 33%)	1) BMI categories 2) BMI and HbA1c 3) BMI and TG 4) sex-stratified BMI	1) Physiological measure (*n* = 1, 17%) 2) T2D complication other than CVD (*n* = 1, 17%) 3) Glucose control (*n* = 4, 67%)	Moderate (*n* = 5, 83%) Very low (*n* = 1, 17%)	^5,25–27,62,63^
Simple: Lipid profile/metabolic syndrome (8 studies) *n* = 23,933 41.0% female 61.7 years	52% European/White, 32% East Asian (Chinese) 16% non-White	Prevalent (*n* = 7, 87.5%) New-onset (*n* = 1, 12.5%) Longitudinal (*n* = 3, 37.5%) Cross-sectional (*n* = 5, 62.5%)	1) Atherogenic dyslipidaemia (y/n) 2) Tertiles of triglyceride-glucose index 3) Quartiles of small dense LDL-cholesterol 4) Triglyceride-glucose index and visceral adiposity 5) Atherogenic index 6)Hypertriglyceridaemic waist phenotype 7) Triglyceride levels 8) Metabolic syndrome (y/n)	1) CVD event (*n* = 3, 37.5%) 2) Physiological measure (brachial-ankle PWV, metabolic syndrome; *n* = 2, 25%) 3) Glucose control (*n* = 1, 12.5%) 4) More than one of the above (*n* = 2, 25%)	Moderate (*n* = 2, 25%) Low (*n* = 4, 50%) Very low (*n* = 2, 25%)	^16–19,64–67^
Simple: OGTT data (4 studies) *n* = 1399 31.0% female 50.7 years	34% White 12% Black 12% Hispanic 39% East Asian (Japanese)	Prevalent (*n* = 1, 25%) New onset (*n* = 3, 75%) Longitudinal (*n* = 2, 50%) Cross-sectional (*n* = 2, 50%)	1) Categorised by fasting and 2 h glucose 2) Treatment assignment and OGTT profile (monophasic, biphasic, and upward curve) 3) OGTT profiles: monophasic curve; biphasic curve & upward curve	1) CVD event (*n* = 1, 25%) 2) Glucose control (*n* = 1, 25%) 3) Physiological measure (glucose response, insulin sensitivity, insulinogenic index; *n* = 2, 50%)	Moderate (*n* = 2, 50%) Low (*n* = 2, 50%)	^28,68–70^
Simple: Alternative approach to stratification (5 studies) *n* = 1,572,942 45.2% female 58.6 years	99.7% East Asian (Korean) 0.28% European/White 0.03% Black	Prevalent (*n* = 4, 80%) New-onset (*n* = 1, 20%) Longitudinal (*n* = 3, 60%) Cross-sectional (*n* = 2, 40%)	1) Total illness burden index 2) Diabetic retinopathy (yes/no) 3) Ketosis-prone defined as new-onset diabetes without precipitating events, 4) Severe hypoglycaemia (yes/no) 5) Diabetes duration	1) CVD event (*n* = 3, 60%) 2) Clinical biomarker (*n* = 1, 20%) 3) Physiological measure (*n* = 1, 20%)	Moderate (*n* = 1, 20%) Low (*n* = 4, 80%)	^48,71–74^
Simple: Cardiovascular features (3 studies) *n* = 8793 53.4% female 62.3 years	75% European/White 16.4% Asian (Chinese, Malay, Indian) 8.6% Black	Prevalent (*n* = 3, 100%) Longitudinal (*n* = 2, 66.6%) Cross-sectional (*n* = 1, 33.3%)	1) Clinic pulse pressure quartiles 2) Pulse wave velocity tertiles 3) Cardiomyopathy (yes/no)	1) CVD event (*n* = 2, 66.6%) 2) T2D complication other than CVD (CKD progression, *n* = 1, 33.3%)	Moderate (*n* = 2, 66.6%) Low (*n* = 1, 33.3)	^75–77^
Simple: Other blood or urine test (11 studies) *n* = 71,609 %47.4 female 62.2 years	70.6% European/White 15.5% East Asian (Chinese, Taiwanese, Japanese) 1.2% Black 0.6% Hispanic 0.1% Middle-Eastern (Egyptian) 11.3% non-White 0.7% Other	Prevalent (*n* = 8, 72.7%) New-onset (*n* = 3, 27.3%) Longitudinal (*n* = 6, 54.5%) Cross-sectional (*n* = 5, 45.5%)	1) UACR ranges (*n* = 2) 2) eGFR (*n* = 1) 3) Glycosuria categories (*n* = 1) 4) C-reactive protein quartiles (*n* = 1) 5) Vaspin and adiponectin (*n* = 1) 6) Quartiles of fibrosis index & albuminuria (*n* = 1) 7) Total bile acid levels (*n* = 1) 8) log(bilirubin) (*n* = 1) 9) Hp2-2 phenotype (*n* = 1) 10) Multiple (HbA1c, total cholesterol, blood pressure) (*n* = 1)	1) Clinical biomarker (*n* = 4, 36.4%) 2) CVD event (*n* = 3, 27.3%) 3) T2D complication other than CVD (*n* = 2, 18.2%) 4) Death (*n* = 2, 18.2%)	Moderate (*n* = 5, 46%) Low (*n* = 3, 27.3%) Very low (*n* = 3, 27.3%)	^78–88^

*T2D* type 2 diabetes, *GAD* glutamic decarboxylase antibody, *UCPCR* urine C-peptide to creatinine ratio, *BMI* body mass index, *CVD* cardiovascular disease, *OGTT* oral glucose tolerance test, *CKD* chronic kidney disease, *LDL* low-density lipoprotein cholesterol.

Different categories of triglycerides, low-density lipoprotein (LDL) cholesterol, high-density lipoprotein (HDL) cholesterol, atherogenic small dense lipoproteins with and without features of metabolic syndrome were used to stratify type 2 diabetes in eight studies. Cardiovascular disease (CVD) outcomes were assessed in 3/8 of the studies^16–18^ which showed that a more atherogenic metric of the specific lipid exposure (e.g., higher LDL cholesterol) was associated with a greater frequency of CVD outcomes. Other outcomes included pulse wave velocity^19^ or clinical characteristics; age, BMI, presence of metabolic syndrome in specific subgroups.

The six studies assessing pancreatic autoantibodies focused on glutamic acid decarboxylase 65 (GAD-65) levels. Studies used positive versus negative status or high versus low titre, and one study sub-stratified by age. Outcomes included time-to-insulin treatment^20,21^, associations with other clinical characteristics such as lipid profiles, BMI and blood pressure^22–24^ and measures of beta-cell function. There was no consistency in study design and most were observational with low to moderate evidence grade; two studies showed that GAD-65 positivity was associated with faster time-to-insulin treatment^20,21^.

Patients with type 2 diabetes were stratified according to their BMI in six studies, either by BMI alone (*n* = 5) or BMI in combination with HbA1c. The number of BMI categories varied between two and six in the identified studies. The association between BMI and glycaemic outcomes (change in HbA1c from baseline) was assessed in four studies either as primary or secondary outcomes^6,25,26^. We graded the quality of evidence as very low to moderate, and no consistency of effect was observed across all studies. In one secondary analysis of a randomised control trial, higher BMI at baseline was associated with faster progression to adverse renal outcomes, however, this was not replicated in any other study^27^.

Age at diagnosis was assessed as a stratification tool in two studies; younger age (mean age 33 years) was associated with higher rates of proliferative retinopathy in an observational study with 12 months follow-up versus older age (mean 50 years)^4^. In a second study, patients aged 60–75 versus those >75 years had a high risk of CVD and mortality when stratified by cholesterol levels^6^. Neither study was replicated to confirm findings.

Four studies used results from oral glucose tolerance tests (OGTT) as exposures. The specific stratification approach applied to OGTT profiles was different in each study and based on cut-offs of fasting glucose levels, glucose gradients after stimulation and responses to different drug treatments. Outcomes included clamp-derived insulin sensitivity and differences in the shape of glucose profiles between youths and adults^28^.

Measures of estimated beta-cell function were assessed in six studies including C-peptide levels and homoeostasis model assessment-2 indices for beta-cell function (HOMA2-B) or insulin resistance (HOMA2-IR), which require measurement of fasting insulin and glucose levels. C-peptide was defined using variable cut-offs. Outcomes included clinical phenotype data, response to medication, and microvascular or macrovascular complications. For example, hyperinsulinaemia and higher urine C-peptide were independently associated with cardiovascular disease.

Other exposure variables included less routine biomarkers, pulse wave velocity, ketosis/ketoacidosis and other disease indices, but these were each single studies precluding grouping. All data are summarised in Table 1.

#### Use of complex approaches to subclassify type 2 diabetes

##### Description of extracted studies

There were 62 studies of complex/ML approaches to type 2 diabetes subclassification in a total of 793,291 participants (Table 2). Over half of the studies included non-European ancestry in relevant proportions (>20%). Only ~30% (19 out of 62) of the studies analysed participants with new-onset diabetes. Mean diabetes duration ranged from recent onset (within 1 year) to over 36 years. Most data were from observational studies (46 out of 62), with some post-hoc analyses of clinical trials (10), survey data (4) and mixed study types (2). Half of the studies had prospective design (31 out of 62) with a mean follow-up duration ranging from 1 year to 11.6 years. K-means clustering was the most applied ML approach (30 out of 62). Eight studies used established centroids^8^ to assign participants to clusters. Two studies decomposed combinations of genetic variants and their association with clinical and laboratory phenotypes into genotype-phenotype clusters by using Bayesian non-negative matrix factorisation.

**Table 2:** Summary of published studies using complex approaches to type 2 diabetes classification.

Approach Total *n* % Female Mean age/age range	% Race/ethnicity breakdown	% Prevalent or new onset T2D, *n* (%) % Prospective or Cross sectional, *n* (%)	Machine learning approach	T2D subclassified Groups identified	Outcome category, *n* (%)	Overall quality, *n* (%)	References
Complex: Ahlqvist and directly replicated Ahlqvist clusters (22 studies) *n* = 88,197 43% female 55.3 years	81% Non-Hispanic White, 11% East Asian, 4% Hispanic, 3% South Asian, <1% Black, <1% Other	Prevalent (*n* = 11, 50%), New onset (*n* = 11, 50%) Longitudinal (*n* = 8, 36.6%), Cross-sectional (*n* = 14, 63.6%)	100% k-means	SAID, SIDD, SIRD, MOD, MARD	Microvascular & macrovascular events (*n* = 9, 41%), Clinical and biochemical traits (*n* = 4, 18%) Microvascular events only (*n* = 3, 13%), Glycaemia (*n* = 2, 9%), Macrovascular events only (*n* = 1, 5%), omic (*n* = 1, 5%), Other (*n* = 2, 9%)	Very Low (*n* = 1, 5%), Low (*n* = 3, 13%), Moderate (*n* = 18, 82%)	^8,40,89–108^
Complex: Similar to Ahlqvist clusters (13 studies) *n* = 214,093 45% female 58.6 years	72% Non-Hispanic White, 12% Hispanic, 10% South Asian, 4% East Asian, 2% Black, <1% Native American, <1% Other	Prevalent (*n* = 11, 85%), New onset (*n* = 2, 15%) Longitudinal (*n* = 7, 54%), Cross sectional (*n* = 6, 46%)	a) Addition of complementary clinical variables (i.e., HDL, TG, waist circumference, uric acid, etc.) b) Incorporating new clustering, i.e., self-normalising neural networks trained on k-means clustering. c) Addition of ethnic-specific thresholds for BMI.	SAID, SIDD, SIRD, MOD, and MARD. Five clusters: Older onset, Severe hyperglycaemia, Severe obesity, Younger Onset, and Insulin use. Four clusters: 42% (older onset), 14% (poor glucose control), 24% (severe obesity), and 20% (younger-onset). New subgroups MD, EOIDD, EOIRD, LOIDD, LOIRD.	Microvascular & macrovascular (*n* = 5, 38%), Microvascular events only (*n* = 3, 23%), Macrovascular events (*n* = 1, 8%), Glycaemia (*n* = 1, 8%), Other (*n* = 3, 23%)	Very Low (*n* = 1, 8%), Low (*n* = 5, 38%) Moderate (*n* = 7, 54%)	^41,52,54,59,109–117^
Complex: Simple clinical features (4 studies) *n* = 22,296 46.5% female 56 years	34% Non-Hispanic White, 25% Asian, 25% Middle Eastern, 9% Black, 5% Hispanic, 1% Native American/American Indian/Alaskan Native, <1% Other	Prevalent T2D (*n* = 3, 75%), New onset (*n* = 1, 25%) Longitudinal (*n* = 3, 75%), Cross-sectional (*n* = 1, 25%)	50% k-means (*n* = 2), 50% other (*n* = 2)	variable	Mortality (*n* = 2, 50%), Cardiovascular events (*n* = 1, 25%), Clinical and biochemical traits (*n* = 1, 25%)	Moderate (*n* = 3, 75%), Low (*n* = 1, 25%)	^118–121^
Complex: Complex clinical features (11 studies) *n* = 386,889 46.8% female 60 years	57% Non-Hispanic White, 24% Asian, 8% Black, 6% Hispanic, <5% Other	Prevalent T2D (*n* = 9, 82%), New onset (*n* = 2, 18%) Longitudinal (*n* = 7, 64%), Cross-sectional (*n* = 4, 36%)	variable	variable	Cardiovascular events (*n* = 4, 36%), Glycaemic control (*n* = 3, 27%), Complications other than CVD event (*n* = 2, 18%), Mortality (*n* = 1, 9%), Other (*n* = 1, 9%)	Moderate (*n* = 9, 82%), Low (*n* = 2, 18%)	^29,30,122–130^
Complex: Cardiovascular features (2 studies) *n* = 974 55.4% female 63 years	100% Non-Hispanic White	Prevalent T2D (*n* = 2, 100%) Longitudinal (*n* = 2, 100%)	Factor analysis (clustering) (*n* = 1, 50%), Hierarchical clustering (*n* = 1, 50%)	3 and 4 clusters	Cardiovascular death and events (*n* = 2, 100%)	Moderate (*n* = 1, 50%), Low (*n* = 1, 50%)	^31,32^
Complex: Behavioural features (2 studies) *n* = 653 40.5% female 63.5 years	48% Non-Hispanic White, 50% Asian, 2% others	Prevalent (n = 1, 50%), New onset (*n* = 1, 50%) Longitudinal (*n* = 1, 50%), Cross sectional (*n* = 1, 50%)	clustering, hierarchical clustering	2 and 4 clusters	Glycaemic control (*n* = 2, 100%)	Moderate (*n* = 1, 50%), Low (*n* = 1, 50%)	^131,132^
Complex: Glycemic features (4 studies) *n* = 67,064 42.8% female 62.5 years	40.6% Non-Hispanic White, 25% Asian, 25% Middle Eastern, 9.4% Other	Prevalent (*n* = 3, 75%), New onset (*n* = 1, 25%) Longitudinal (*n* = 3, 75%), Cross sectional (*n* = 1, 25%)	50% k-means, 25% latent-class analysis, 25% hierarchical clustering	3 and 4 clusters	Glycaemic control (*n* = 2, 50%), Cardiovascular events (*n* = 2, 50%)	Moderate (*n* = 3, 75%), Very low (*n* = 1, 25%)	^33–35,133^
Complex: Genetics (3 studies) *n* = 42,952	100% Non-Hispanic White	Prevalent T2D (*n* = 3, 100%) Cross sectional (*n* = 3, 100%)	Bayesian Non-negative Matrix Factorisation (n = 2, 67%), Hierarchical clustering (*n* = 1, 33%)	5 clusters of variant-trait associations; 3 clusters of skeletal dysregulated genes/pathways in people with diabetes	Coronary artery disease, stroke, renal disease	Moderate (*n* = 2, 67%), Low (*n* = 1, 33%)	^10,37,38^
Complex: Hormonal (1 study) *n* = 96 participants 53% female 62 years	100% Non-Hispanic White	New onset (*n* = 1, 100%) Cross sectional (*n* = 1, 100%)	Two-step cluster analysis using log-likelihood distance measures	Two clusters (cluster 1: low GLP-1 and Ghrelin; cluster 2: high GLP-1 and Ghrelin)	Glycemia (*n* = 1, 100%)	Moderate (*n* = 1, 100%)	^36^

*T2D* type 2 diabetes, *GAD* glutamic decarboxylase antibody, *UCPCR* urine C-peptide to creatinine ratio, *BMI* body mass index, *CVD* cardiovascular disease, *OGTT* oral glucose tolerance test, *CKD* chronic kidney disease, *LDL* low density lipoprotein cholesterol, *SAID* severe autoimmune diabetes, *SIDD* severe insulin deficient diabetes, *SIRD* severe insulin resistant diabetes, *MOD* mild obesity-related diabetes, *MARD* mild age-related diabetes, *MD* mild diabetes, *EOIDD* early-onset insulin deficient diabetes, *EOIRD* early-onset insulin resistant diabetes, *LOIDD* late-onset insulin deficient diabetes, *LOIRD* late-onset insulin resistant diabetes.

##### Description of the categorised subgroups

Following the seminal work by Ahlqvist et al.^8^, multiple studies used the variables derived at time of diabetes diagnosis: age, HbA1c, BMI, HOMA2-B, HOMA2-IR and GAD-65 antibody (Table 2). The majority of these studies employed C-peptide-based homoeostasis model assessment indices (HOMA, or its updated variant, HOMA2, using fasting insulin and glucose), as surrogates for insulin resistance (HOMA2-IR) and insulin secretion (HOMA2-B). In different contexts and populations, 22 studies replicated identification of the four non-autoimmune diabetes subtypes first described by Ahlqvist et al.^8^: severe insulin-deficient diabetes (SIDD), severe insulin-resistant diabetes (SIRD), mild obesity-related diabetes (MOD), and mild age-related diabetes (MARD). The subset of studies including measurements of GAD antibody also identified the fifth cluster, severe autoimmune diabetes (SAID). Associations of these subtypes with clinical outcomes, including glycaemia, microvascular and macrovascular outcomes, and death, were replicated in 12 studies (Table 3).

**Table 3:** Association of the Ahlqvist-clusters with outcomes from 22 reviewed studies using consistent cluster assignment methods.

	Glycemic outcomes	Microvascular outcomes	Macrovascular outcomes	Other outcomes	Death
SIDD	Insulin requirement^8,97^	Retinopathy^8,97,102–104^ (*n* = 3160) Nephropathy^97,103^ (*n* = 1074)	Lower extremity arterial disease^103^ (*n* = 223)	Distal symmetric polyneuropathy, Cardiac autonomic neuropathy^90^ (*n* = 28), Erectile dysfunction^93^ (*n* = 4)	
SIRD	Glycemic benefit with thiazolidinedione therapy^40^ (*n* = 800)	Diabetic kidney disease^8,90,98,100,103,107^ (*n* = 2686)	MACE (confounded by age and sex^8^, *n* = 1373)	Prevalence of NAFLD^90^ (n = 121), Erectile dysfunction^93^ (*n* = 7), Diabetic peripheral neuropathy^103^ (*n* = 225)	
MOD				Neuropathy^97^ (*n* = 1258)	
MARD	Glycemic benefit with sulfonylurea therapy^40^ (*n* = 1361)	Retinopathy^100^(*n* = 1487)	MACE^8,97^ (*n* = 3513)		CVD mortality^102^ (*n* = 56)

*SAID* severe autoimmune diabetes, *SIDD* severe insulin deficient diabetes, *SIRD* severe insulin resistant diabetes, *MOD* mild obesity-related diabetes, *MARD* mild age-related diabetes.

**Fig. 3:**
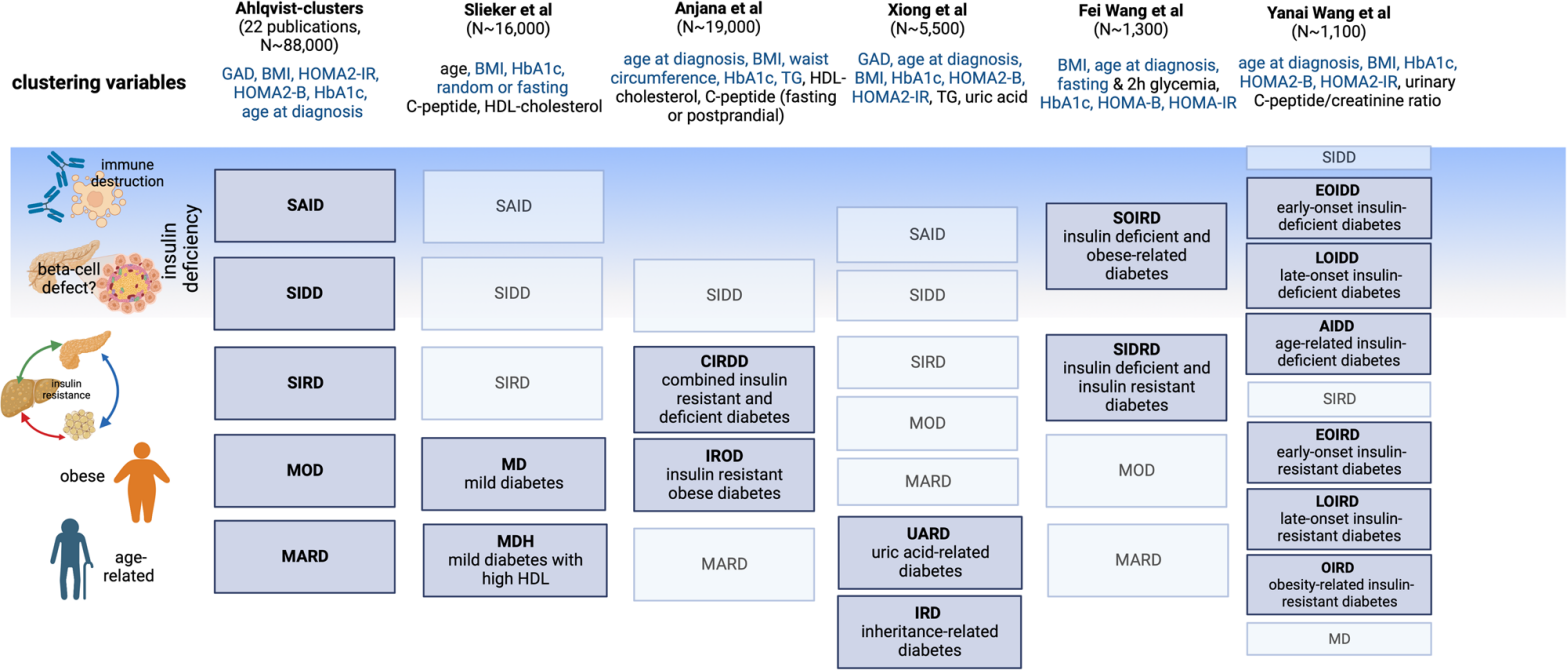
Main characteristics of diabetes clusters derived using a modified set of clustering variables, compared to original ‘Alhqvist’ clusters. Clustering variables denoted in blue are consistent across the different studies, those in black are unique to the particular study outlined. A greyed-out box indicates that the indicated diabetes cluster was replicated from the Ahlqvist study, a dark blue box indicates a new diabetes cluster. GAD, glutamic decarboxylase antibody; BMI, body mass index; HDL, high-density lipoprotein cholesterol; HOMA2-IR/B, homoeostasis model assessment-2 insulin resistance/beta cell function. SAID, severe autoimmune diabetes; SIDD, severe insulin-deficient diabetes; SIRD, severe insulin resistant diabetes; MOD, mild obesity-related diabetes; MARD, mild age-related diabetes.

Thirteen additional papers used variations of the original set of variables from Ahlqvist et al.^8^ by substituting HOMA with C-peptide, adding lipid traits, e.g. HDL-cholesterol, or approximating the clusters from different/simplified variable sets by applying advanced statistical learning approaches such as self-normalising neural networks. These approaches identified some type 2 diabetes subgroups resembling the clusters from Ahlqvist et al. and also novel subgroups related to the additional variables (Fig. 3). Several of the novel subgroups were associated with clinical outcomes. However, these findings have not been replicated in other studies (Table 2).

Additional papers (*n* = 27) assessed various sets of phenotypic inputs for ML approaches. Grouped into five categories of inputs, studies identified many subtypes and associations with clinical outcomes, however, they all lacked replication (Table 2). Four papers applied complex ML methods to a set of less than ten clinical variables such as systolic blood pressure, waist circumference, BMI, fasting plasma glucose, and age at diabetes diagnosis, and resulting subgroups were variably associated with outcomes, such as mortality. Eleven studies used a larger set of more than ten clinical features as inputs for classification, including data from electronic health records^29,30^, and identified subgroups variably associated with clinical outcomes, including risk of cardiovascular disease. Two other studies specifically employed cardiovascular traits, including ECG^31^ and echocardiographic^32^ for ML algorithm inputs, and each identified subgroups with different associations with risk of cardiovascular disease. Finally, four studies involved inputs of change of glycaemic variables (HbA1c trajectories, glycaemia during a mixed meal test, continuous glucose monitoring features)^33–35^, one study focused on fasting GLP-1, GIP and ghrelin levels^36^, and two studies focused on behavioural traits such as novelty seeking, harm avoidance, and hospital anxiety and depression scale.

Human genetic risk information is rapidly penetrating clinical medicine. Two sets of papers utilised genomic data to identify diabetes subtypes, either in the form of inherited common genetic variation^10,37^ or gene expression data from muscle biopsies^38^ (Table 2). The first approach clustered genetic variants with clinical traits associated with type 2 diabetes to identify subsets of variants predicted to act in shared mechanistic processes. Using these sets of genetic variants, process-specific or partitioned polygenic scores were constructed in individuals with type 2 diabetes and were associated with differences in clinical features and prevalence of metabolic outcomes, with replication across multiple cohorts. The muscle gene expression study has not been replicated. Overall, half of the studies had cross-sectional designs, and the other half involved prospective follow-up (Table 2).

#### Quality assessment

For simple approaches, of the 51 studies assessed, 55% were quality graded as very low-, or low-GRADE certainty, 45% had moderate certainty and none achieved high certainty. For complex approaches, around 70% of the studies had moderate evidence certainty. In both approaches, the majority of the studies had moderate or lower GRADE certainty on account of the (1) study design not addressing precision medicine objectives (not an RCT testing differential treatment effects in subclassified type 2 diabetes groups), (2) lack of a meaningful clinical outcome (i.e. although subgroups of type 2 diabetes were found, the measured outcome had little clinical significance because the study was not designed to study this) (3) Confidence in the findings were low due to small sample sizes, lack of replication or lack of diversity of studied subgroups and (4) the potential for bias was large due to lack of adjustment for possible confounders.

## Discussion

### Summary of findings

This systematic review analysed two broad approaches to the subclassification of type 2 diabetes to identify clinically meaningful subtypes that may advance precision diagnostics. We found many simple stratification approaches using, for example, clinical features such as BMI, age at diagnosis, and lipid levels, but none had been replicated and many lacked associations with clinical outcomes. Complex stratification models using ML approaches with and without genetic data showed reproducible subtypes of type 2 diabetes associated with outcomes. Both approaches require a higher grade of evidence but support the premise that type 2 diabetes can be subclassified into clinically meaningful subtypes.

Simple approaches to subclassification included urine and blood biomarkers, anthropometric measures, clinical data such as age at diagnosis, surrogate beta-cell metrics derived from blood C-peptide or insulin along with other less diabetes-related biomarkers such as bilirubin levels or pulse wave velocity. Approaches to subclassification were diverse. Some studies dichotomised continuous variables based on clinical cut-points. Other studies used a composite exposure (two or more criteria each with cut-points) or analysed changes in continuous variables over time e.g. change in eGFR over time.

The study designs, specific cut-offs and outcomes were heterogenous, and no studies met high-quality GRADE certainty. No study evaluating a simple approach to type 2 diabetes subtyping has been adequately reproduced, although some studies identified biologically plausible subgroups. For example, subclassifications derived using BMI, beta-cell function, lipid profiles and age appeared to be associated with some outcomes which could be helpful in clinical practice. These potential subclassifications need to be replicated in better-designed studies (see section on additional supporting literature). Other evidence not included in our systematic review (either due to the study population including people without diabetes or the analysis was only performed in people with the exposure without a comparison group), support the role of simple variables in stratifying diabetes; for example, younger age at diagnosis is reproducibly associated with worse cardiorenal outcomes in a number of studies^39^.

Machine learning approaches yielded some reproducible subtypes of type 2 diabetes using a variety of clinical and genetic variables. The best-replicated subtypes were the clusters first described by Ahlqvist et al.^8^, which were replicated in 22 studies, including ~88,000 individuals of diverse ancestry. There also was replication of genetic subtypes of type 2 diabetes from Udler et al.^10^ with associations with clinical features seen in multiple cohorts across almost 454,000 individuals^36^. However, the latter associations involved small absolute effects with unclear clinical utility for individual patient management, and studies were restricted to individuals of European ancestry. While there was replication of the clusters from Ahlqvist et al. across studies, the generated clusters appeared to be dependent on the characteristics of the underlying populations, especially factors such as distribution of ancestry, age, duration of diabetes, anthropometric trait variability as in BMI, and the variety of variable terms included in learning models. Nevertheless, at least some of the resulting subtypes appeared to be robust to differences in specific ML method, input variables, and populations (Fig. 3).

Many of the input variables for the complex ML subtyping approaches were also used in studies involving simple approaches to subclassification, recapitulating the biological plausibility of specific clustering variables in defining type 2 diabetes subtypes. One study directly compared a simple clinical approach to the clustering approach from Ahlqvist et al.^8^ and found that simple single clinical measures analysed in a quantitative (rather than categorical) framework could better predict relevant clinical outcomes, such as incidence of chronic kidney disease and glycaemic response to medications^40^. Thus, further research is needed to determine whether assigning a patient to one of the clusters from Ahlqvist et al.^8^ offers additional clinical benefit beyond evaluation of simple clinical measures and also beyond current standard of care. For example, high quality randomised controlled trial evidence is needed to demonstrate that knowledge of a patient’s clinical or genetic cluster membership could meaningfully guide treatment and/or clinical care and improve outcomes.

### Study quality

No studies included in our systematic review had above moderate certainty of evidence. Some strengths of included studies were the large sample sizes, the diversity of variables considered, and inclusion of both prevalent and new-onset cases of type 2 diabetes. However, the varied study designs and lack of replication limits our ability to draw firm conclusions about the most effective approaches to subclassification. Most variables used for subclassification capture momentary metabolic states, which limits their long-term utility as cluster assignment is likely to change over time^41,42^. Most studies were retrospective analyses of established cohorts, and there were, at the time of the search, no data available involving subtype-stratified clinical trials or real world implementation of approaches. Finally, most studies focused on European-ancestry populations, and the clinical value of these approaches may vary across different ancestries. While East Asian ancestries had representation in some studies, research in Black, South Asian and Hispanic populations remains sparse. This is particularly important, as four out of five people with type 2 diabetes come from marginalised groups or live in low- or middle-income countries. Future precision diagnostic interventions should address and narrow inequalities.

### Additional supporting literature

Since our literature search was conducted, four new publications have advanced our understanding of type 2 diabetes subclassification.

Two recent studies applied ML approaches to stratify diabetes heterogeneity, both considering continuous approaches rather than with discrete clusters^43,44^. Nair et al. used a non-linear transformation and visualisation of nine variables onto a tree-like structure^44^ and with replication in two large datasets. This approach linked underlying disease heterogeneity to risk of complications; those at risk of cardiovascular disease had a different phenotype to those with microvascular complications and to drug response and demonstrated associations of gradients across the tree using genetic process-specific scores from Udler et al.^10^ Wesolowska-Andersen et al. performed soft-clustering from 32 clinical variables which yielded 4 diabetes archetypes comprising a third of the study population. The remaining study population was deemed as mixed-phenotype. This study has not been replicated^43^. A third study re-identified the genetic subtypes and their clinical associations from Udler et al.^45^.

Additionally, one of the first clinical trials to assess precision medicine approaches for diabetes management was published after our literature search. The TriMaster Study tested dichotomised BMI and eGFR strata in a three-period crossover trial using three pharmacologic interventions with the primary hypothesis being stratum-specific differences in HbA1c^46^. Participants with obesity (BMI > 30 kg/m^2^) showed a glycaemic benefit on pioglitazone versus sitagliptin and participants with lower eGFR (60–90 ml/min/1.73 m^2^) responded with lower HbA1c to sitagliptin as compared to canagliflozin. In a secondary analysis, drug-choice corresponding to patient preferences yielded lower glycemia than a random allocation, suggesting that listening to patients is critical in informing therapeutic decisions^47^. Ramifications of this study are limited by the non-comparable pharmacologic doses used, and the primary focus on glycaemia which may not be indicative of long-term therapeutic success and/or prevention of complications. Yet these studies have generated higher quality evidence linking type 2 diabetes heterogeneity to treatment and disease outcomes. It remains to be seen if these can be replicated in other ancestries and translated into ‘usable products’ for healthcare professionals.

It is worth noting that ketosis-prone type 2 diabetes, an established type 2 diabetes subtype, was not captured adequately in our systematic review: only one study included ketosis-prone type 2 diabetes as an exposure^48^. Study designs for ketosis-prone type 2 diabetes were usually analyses of cohorts with diabetic ketoacidosis at presentation with type 1 diabetes as the outcome, rather than as an exposure in people with type 2 diabetes. Since our search was designed to identify studies stratifying type 2 diabetes, this literature was not captured. Like many other ‘simple’ criteria for classification, the characteristics of people with diabetic ketoacidosis at presentation of type 2 diabetes have been studied, but with few prospective studies that have been replicated^49^.

Age at diagnosis as a simple approach to stratification also did not feature strongly in our search results. The body of literature that outlines higher risk of microvascular or macrovascular complications in early-onset type 2 diabetes has focussed on comparing people with type 2 diabetes to those without diabetes in different age groups^39,50^ or studied cohorts of early-onset cases in isolation^51^ and, thus, would not have been captured in our search strategy. Recent epidemiological studies have compared outcomes between early and late age onset strata^52,53^ showcasing higher risks of cardiorenal outcomes with early age at onset, but these were retrospective analyses of health record databases, potentially confounded by age-related risk of complications and duration of diabetes. To move forward, prospective studies stratifying different interventions (e.g., tighter treatment targets or better cardiovascular risk reduction) in those diagnosed at younger age, are needed.

### Findings in context

We found that simple features have not been precisely and reproducibly evaluated to a high enough standard to subclassify type 2 diabetes into subtypes. This is not surprising, as many studies were not necessarily conducted for the purpose of ‘precision diagnosis’, but rather as studies of clinical phenotypes spanning a time period that preceded the current research focus on precision medicine. It is important to re-emphasise that many of the simple clinical criteria studied, do have other bodies of evidence supporting associations with outcomes, like age -at -diagnosis. While these studies have set the scene, the field needs more robust evidence.

‘Complex’ methods for diabetes subclassification have shown better reproducibility, have been linked to a variety of meaningful clinical outcomes more consistently, and more recently have been able to demonstrate differential treatment responses related to stratification.

What do these findings mean for a precision medicine approach to type 2 diabetes diagnosis? Ideally, subclassification strategies should be deployed at diagnosis of type 2 diabetes on the basis of measured clinical characteristics such that people in different subgroups of type 2 diabetes could be treated differently. One key question is whether such efforts would cost-effectively improve clinical outcomes, compared to the current standard of care. However, another more fundamental question is whether subclassification approaches at diagnosis alone are enough? For example, another approach may be to iteratively subclassify longitudinal disease trajectories. Such an approach is supported by studies that have shown cluster-based assignments of type 2 diabetes at diagnosis are not robust and may change over time^54^. It may be argued that subclassification at one-time point is overly simplistic and should be regularly reviewed based on trajectory.

Irrespective of the subclassification approach studied, they need replication in independent datasets, assessment in diverse populations, in people with both new-onset and prevalent diabetes, and investigation using prospective data, ideally in the form of randomised clinical trials. Clinical trials of treatment approaches tailored to diabetes subtypes will be necessary to understand the clinical benefits of clinical subtyping. Ideally, sub-phenotyping should lead to benefits for patients in real-world clinical settings. Conducting these studies will be challenging due to the necessity for extensive follow-up, large sample sizes, and substantial resource requirements. There is a pressing need for innovative strategies to generate high-quality evidence on treatment options tailored to specific diabetes subtypes in diverse populations. These data will be critical to determine generalisability of findings and amenability for clinical translation including in resource-constrained settings.

### Clinical applicability

The current evidence supports distinguishable subtypes of type 2 diabetes and that these subtypes are associated with variation in clinical outcomes. However, the very low to moderate quality of existing studies and the need for replication in ancestry-diverse studies make it difficult to identify a strongly evidence-based, universally applicable approach.

The most clinically valuable methods are likely to be those that are easy and inexpensive to implement. For more complex approaches, computer decision support tools will need to be developed and assessed for feasibility and utility. Although the evidence supporting complex approaches has leap-frogged the evidence in favour of more simplified approaches, there is still likely a place for simple approaches that can be more accessible at diverse clinical interfaces. Meanwhile how cluster assignment could be translated into actionable data for the individual remains unclear; will for example, a given person with type 2 diabetes exist in a distinct subgroup with associated outcomes or will the subtype of type 2 diabetes have associated probabilities or risks of certain outcomes? While stratifying people with type 2 diabetes into discrete subtypes might result in information loss, compared to continuous risk modelling^40^, discrete clusters might inform clinical decisions^42^.

### Limitations

The limitations of this review reflect the limitations of the literature. To manage the breadth of literature analysed in this systematic review, focussed on genomic data and did not include proteomic or metabolomic data as these are potentially more premature for clinical use. We also did not include studies on participants at risk of type 2 diabetes, although we recognise that a body of evidence is emerging to stratify type 2 diabetes incidence risk using multiple approaches that are similar to those for established type 2 diabetes. Since we focused on studies that attempted to subgroup type 2 diabetes, we also did not capture analyses of independent cohorts with a particular type 2 diabetes phenotype at baseline, for example, studies of young people with type 2 diabetes or those with ketosis-prone type 2 diabetes, as outlined.

### Next steps and recommendations

Future research should aim to identify and validate clinically useful and cost-effective methods for type 2 diabetes subclassification that can be applied across diverse populations. Such research will involve replication of a given approach in independent datasets, including from diverse ancestral populations, to ensure generalisability that doesn’t widen health disparities. For simple stratification approaches, there is still much that can be done—agreement on standardised study designs for precision diagnostics studies could be a first step. For ML requiring real-time computation, the development of strategies to overcome local resource constraints in implementing these methods could be explored.

## Conclusion

In this first systematic review of the evidence underpinning type 2 diabetes diagnostic subclassification, multiple approaches were identified. Among them are strategies that used simple criteria based on fundamental categorisation of mostly routine measures, and complex approaches with multi-trait or genetic inputs that required ML or other computation. While simple approaches are more easily deployed, the study designs and level of evidence currently limits any firm conclusions regarding the utility of such approaches. The clinical variables and data incorporated into ‘complex’ approaches have yielded reproducible subclassifications and a growing body of evidence supports clinically meaningful associations of subtypes with outcomes and treatment responses. This is a rapidly evolving field with higher quality evidence emerging. It will be crucial to develop interventions that target diverse populations and be feasible in all resource settings to prevent widening existing inequalities in the precision medicine era of diabetes care.

### Supplementary information

Supplementary information

Supplementary Data 1

Description of Additional Supplementary Files

Reporting Summary

## Data Availability

The extracted data from full-text articles included in this systematic review are available in Supplementary Data 1.
