# Supplementary material for: Precision subclassification of type 2 diabetes: a systematic review
Source: Commun Med (Lond). 2023 Oct 5;3:138. doi: 10.1038/s43856-023-00360-3 (PMC10556101; doi:10.1038/s43856-023-00360-3)
Supplement: Supplementary file 1 — Supplementary information [file 43856_2023_360_MOESM1_ESM.pdf]

Supplementary Table 1: PICO framework used in systematic review

**Criteria**	**Determinant**
Population	Studies reporting on patients with incident or prevalent type 2 diabetes
Exposure	Any approach taken to classifying people with type 2 diabetes using search terms including "classification", "subtype", "category", "phenotype", "precision medicine", "heterogeneity" (see supp table).<br><br>There were no restrictions on the approach used e.g. biomarker, anthropometry, imaging and any others
Comparison	Subgroups of type 2 diabetes identified following classification using the exposure were compared
Outcome	Any outcome in the groups were accepted e.g phenotype data, glycaemia, microvascular complications, macrovascular complications, mortality and others
Study Designs	All designs were accepted

Supplementary methods

Question 1 Search Terms (Simple approaches to classification)

#1
"Diabetes Mellitus, Type 2"[Mesh]
=154385

#2
type 2 diabetes[Title/Abstract] OR niddm[Title/Abstract] OR t2dm[Title/Abstract] OR t2d[Title/Abstract] OR "noninsulin dependent diabetes"[Title/Abstract] OR "non insulin dependent diabetes"[Title/Abstract] OR "type 2"[Title/Abstract] OR "type ii"[Title/Abstract] OR type2[Title/Abstract] OR typeii[Title/Abstract] OR adult-onset diabetes[Title/Abstract] OR prediabetic[Title/Abstract] OR pre-diabetic[Title/Abstract] OR Non-insulin dependent diabetes mellitus
=378950

#3
#1 OR #2
=378950

#4
"Classification"[Mesh] OR "classification" [Subheading]
=753381

#5
classificat*[Title/Abstract] OR sub classificat*[Title/Abstract] OR subclassificat*[Title/Abstract] OR subgroup*[Title/Abstract] OR "sub-group*'"OR subtype*[Title/Abstract] OR "glucose response curve"[Title/Abstract] OR "body shape index"[Title/Abstract] OR "fat evaluation"[Title/Abstract] OR "fasting insulin resistance index"[Title/Abstract] OR metabolic basis[Title/Abstract] OR immunologic basis[Title/Abstract] OR immunological basis[Title/Abstract]
=850738

#6
#4 OR #5
=1494817

#7
((("Precision Medicine"[Mesh]) OR "Genetic Heterogeneity"[Mesh]) OR "Phenotype"[Mesh]) OR "Blood Glucose"[Mesh]
=535079

#8

"precision medicine"[Title/Abstract] OR heterogeneity[Title/Abstract] OR variability[Title/Abstract] OR phenotyp*[Title/Abstract] OR pattern*[Title/Abstract] OR severity[Title/Abstract] OR feature*[Title/Abstract] OR characteristic*[Title/Abstract] OR predict*[Title/Abstract] OR stage*[Title/Abstract] OR glycemia[Title/Abstract] OR glycaemia[Title/Abstract] OR "perzonalised medicine"[Title/Abstract] OR "personalised medicine"[Title/Abstract] OR forecast*[Title/Abstract] OR determinant*[Title/Abstract]
=7227358

#9
#7 OR #8
=7484521

#10
"Medical History Taking"[Mesh]
=22396

#11
family history[Title/Abstract] OR relative[Title/Abstract]
=993615

#12
#10 OR #11
=1013777

#13
#9 OR #12
=8116688

#14

((((((((((((("Insulin-Secreting Cells"[Mesh]) OR "Islets of Langerhans"[Mesh]) OR "C-Peptide"[Mesh]) OR "Glucose Tolerance Test"[Mesh]) OR "Glucose Clamp Technique"[Mesh]) OR "Biomarkers"[Mesh]) OR ("Body Mass Index"[Mesh] OR "Obesity"[Mesh] OR "Overweight"[Mesh])) OR "Insulin Resistance"[Mesh]) OR "Body Weight"[Mesh]) OR "Glycemic Index"[Mesh]) OR "Metabolic Syndrome"[Mesh]) OR "Lipids"[Mesh]) OR "Cholesterol"[Mesh]) OR "Dyslipidemias"[Mesh]) OR "Body Fat Distribution"[Mesh]) OR "Intra-Abdominal Fat"[Mesh]
=2638388

#15
Beta cell*[Title/Abstract] OR betacell*[Title/Abstract] OR beta-cell*[Title/Abstract] OR beta cell dysfunction[Title/Abstract] OR beta cell function[Title/Abstract] OR insulin secretion[Title/Abstract] OR c-peptide[Title/Abstract] OR "first phase"[Title/Abstract] OR "glucose tolerance test"[Title/Abstract] OR ogtt[Title/Abstract] OR ivgtt[Title/Abstract] OR clamp[Title/Abstract] OR biomarker*[Title/Abstract] OR biological marker*[Title/Abstract] OR "body mass index"[Title/Abstract] OR bmi[Title/Abstract] OR over weight[Title/Abstract] OR overweight[Title/Abstract] OR obesity[Title/Abstract] OR adiposity[Title/Abstract] OR "insulin resistance"[Title/Abstract] OR homa[Title/Abstract] OR homeostasis model assessment[Title/Abstract] OR weight[Title/Abstract] OR score[Title/Abstract] OR

index[Title/Abstract] OR metabolic syndrome[Title/Abstract] OR excess fat mass[Title/Abstract] OR lipid*[Title/Abstract] OR triglycerid*[Title/Abstract] OR cholesterol[Title/Abstract] OR dyslipidemia[Title/Abstract] OR body fat distribution[Title/Abstract] OR bodyfat distribution[Title/Abstract] OR ketosis[Title/Abstract] OR atypical[Title/Abstract] OR "age at onset"[Title/Abstract] OR "age at diagnosis"[Title/Abstract] OR ketoacidosis[Title/Abstract]
=3645559

#16
#14 OR #15
=5129710

#17
#3 AND #6 AND #13 AND #17
=7208

#18
#17 Filters:Humans
=5993

#19
(review[Publication Type] OR "systematic review"[Publication Type] OR (editorial[Title/Abstract] OR review[Title/Abstract])
=2902309

#20
#18 NOT #19
=**4825 references**

Question 2 Search Terms (Complex approaches to classification)

#1
Diabetes mellitus/classification[MeSH terms] OR diabetes mellitus type 2/classification[Mesh Terms] OR diabetes mellitus type 2/epidemiology[MeSH Terms] OR diabetes mellitus type 2/physiopathology[MeSH Terms] OR diabetes mellitus type 2[MeSH Terms] OR diabetes complications[MeSH Terms] OR Prediabetes[MeSH Terms]
=280913

#2
"Diabetes mellitus, type 2"[Title/Abstract] OR T2D[Title/Abstract] OR "diabetes mellitus type-2" prediabetes[Title/Abstract] OR pre-diabetes IGT[Title/Abstract] OR IFC[Title/Abstract] OR type 2 diabetes[Title/Abstract] OR cluster based diabetes[Title/Abstract] OR cluster-based diabetes[Title/Abstract] OR "adult onset diabetes"[Title/Abstract] OR adult-onset diabetes[Title/Abstract] Filters: Humans
=116423

#3
"adult-onset diabetes subgroups"

=5716

#4
#1 OR #2 OR #3
=302899

#5
((((("Phenotype"[Mesh]) OR "Body Mass Index"[Mesh]) OR "Autoantibodies"[Mesh]) OR "Blood Glucose"[Mesh]) OR "Glycated Hemoglobin A"[Mesh]) OR "Hypoglycemic Agents"[Mesh]
=809305

#6
(((((("Genetic Loci"[Mesh]) OR "Genetic Markers"[Mesh]) OR "Genetic Predisposition to Disease"[Mesh]) OR "Genome-Wide Association Study"[Mesh]) OR "Bayes Theorem"[Mesh]) OR "Founder Effect"[Mesh]) OR "Multigene Family"[Mesh]
=366199

#7
classification[Title/Abstract] OR phenotyp*[Title/Abstract] OR subphenotyp*[Title/Abstract] OR subtype*[Title/Abstract] OR "clinical type*"[Title/Abstract] OR autoantibod*[Title/Abstract] OR "blood glucose"[Title/Abstract] OR "glycated hemoglobin A"[Title/Abstract] OR "genetic loci"[Title/Abstract] OR "genetic predisposition to disease"[Title/Abstract] OR "genome-wide association study"[Title/Abstract] OR "founder effect*"[Title/Abstract] OR "bayes theorem"[Title/Abstract] OR HOMA*[Title/Abstract] OR subgroup*[Title/Abstract] OR HbA1c[Title/Abstract] OR A1C[Title/Abstract]
=1681470

#8
#5 OR #6 OR #7
=2391509

#9
((("Cluster Analysis"[Mesh]) OR ("Supervised Machine Learning"[Mesh] OR "Unsupervised Machine Learning"[Mesh] OR "Machine Learning"[Mesh])) OR "Artificial Intelligence"[Mesh]) OR "Monitoring, Physiologic"[Mesh]
=398710

#10
cluster analysis[Title/Abstract] OR machine learning[Title/Abstract] OR reinforcement learning[Title/Abstract] OR deep learning[Title/Abstract] OR CNN[Title/Abstract] OR "convolutional neural network"[Title/Abstract] OR "data driven cluster*"[Title/Abstract] OR "data-driven cluster*"[Title/Abstract] OR "physiologic monitor*"[Title/Abstract] OR "artificial intelligence"[Title/Abstract] OR AI[Title/Abstract] OR clusters[Title/Abstract] OR subgroup*[Title] OR subtype*[Title]
=365087

#11
#9 OR #10

=689538

#12
#4 AND #8 AND #11
=5986
