# Supplementary material for: Precision subclassification of type 2 diabetes: a systematic review
Source: Commun Med (Lond). 2023 Oct 5;3:138. doi: 10.1038/s43856-023-00360-3 (PMC10556101; doi:10.1038/s43856-023-00360-3)
Supplement: Supplementary file 3 — Description of Additional Supplementary Files [file 43856_2023_360_MOESM3_ESM.pdf]

Description of Additional Supplementary Files

File Name: Supplementary Data 1

Description: Complete data extract of all full-text papers reviewed for both simple and complex approaches
