# Supplementary material for: Precision subclassification of type 2 diabetes: a systematic review
Source: Commun Med (Lond). 2023 Oct 5;3:138. doi: 10.1038/s43856-023-00360-3 (PMC10556101; doi:10.1038/s43856-023-00360-3)
Supplement: Supplementary file 4 — Reporting Summary [file 43856_2023_360_MOESM4_ESM.pdf]

nature portfolio

Corresponding author(s): Shivani Misra

Last updated by author(s): 2023/08/02

Reporting Summary

Nature Portfolio wishes to improve the reproducibility of the work that we publish. This form provides structure for consistency and transparency in reporting. For further information on Nature Portfolio policies, see our Editorial Policies and the Editorial Policy Checklist.

Statistics

For all statistical analyses, confirm that the following items are present in the figure legend, table legend, main text, or Methods section.

n/a	Confirmed	
☒	☐	The exact sample size (*n*) for each experimental group/condition, given as a discrete number and unit of measurement
☒	☐	A statement on whether measurements were taken from distinct samples or whether the same sample was measured repeatedly
☒	☐	The statistical test(s) used AND whether they are one- or two-sided<br>*Only common tests should be described solely by name; describe more complex techniques in the Methods section.*
☒	☐	A description of all covariates tested
☒	☐	A description of any assumptions or corrections, such as tests of normality and adjustment for multiple comparisons
☒	☐	A full description of the statistical parameters including central tendency (e.g. means) or other basic estimates (e.g. regression coefficient) AND variation (e.g. standard deviation) or associated estimates of uncertainty (e.g. confidence intervals)
☒	☐	For null hypothesis testing, the test statistic (e.g. *F*, *t*, *r*) with confidence intervals, effect sizes, degrees of freedom and *P* value noted<br>*Give P values as exact values whenever suitable.*
☒	☐	For Bayesian analysis, information on the choice of priors and Markov chain Monte Carlo settings
☒	☐	For hierarchical and complex designs, identification of the appropriate level for tests and full reporting of outcomes
☒	☐	Estimates of effect sizes (e.g. Cohen's *d*, Pearson's *r*), indicating how they were calculated

Our web collection on statistics for biologists contains articles on many of the points above.

Software and code

Policy information about availability of computer code

Data collection	We used COVIDENCE a proprietary systematic review software to undertake our extraction
Data analysis	No software was used

For manuscripts utilizing custom algorithms or software that are central to the research but not yet described in published literature, software must be made available to editors and reviewers. We strongly encourage code deposition in a community repository (e.g. GitHub). See the Nature Portfolio guidelines for submitting code & software for further information.

Data

Policy information about availability of data

All manuscripts must include a data availability statement. This statement should provide the following information, where applicable:

- Accession codes, unique identifiers, or web links for publicly available datasets
- A description of any restrictions on data availability
- For clinical datasets or third party data, please ensure that the statement adheres to our policy

The extracted data from full-text articles included in this systematic review are available in a data supplement to this manuscript.

Human research participants

Policy information about studies involving human research participants and Sex and Gender in Research.

Reporting on sex and gender	In our synthesis of data from systematic review of relevant studies, we have used sex and stated proportions of male and females.
Population characteristics	All populations studied are described according to ethnicity, age and geographical location.
Recruitment	This was not relevant to the systematic review undertaken
Ethics oversight	Ethics approval was not needed as this was a systematic review

Note that full information on the approval of the study protocol must also be provided in the manuscript.

Field-specific reporting

Please select the one below that is the best fit for your research. If you are not sure, read the appropriate sections before making your selection.

☒ Life sciences ☐ Behavioural & social sciences ☐ Ecological, evolutionary & environmental sciences

For a reference copy of the document with all sections, see nature.com/documents/nr-reporting-summary-flat.pdf

Life sciences study design

All studies must disclose on these points even when the disclosure is negative.

Sample size	Sample size calculation was not necessary for this systematic review nut we have reported sample sizes of all the studies included
Data exclusions	We excluded papers not relevant to our systematic review questions. Broadly these were studies that did not involve populations of people with incident or prevalent type 2 diabetes or lack of stratification approach
Replication	We sought to ascertain whether studies were replicated and reported these in the results
Randomization	Some of the full-text articles included did randomise participants however the method of randomisation was not deemed relevant to our systematic review
Blinding	this was not relevant to our study design

Reporting for specific materials, systems and methods

We require information from authors about some types of materials, experimental systems and methods used in many studies. Here, indicate whether each material, system or method listed is relevant to your study. If you are not sure if a list item applies to your research, read the appropriate section before selecting a response.

Materials & experimental systems

n/a	Involved in the study	
☒	☐	Antibodies
☒	☐	Eukaryotic cell lines
☒	☐	Palaeontology and archaeology
☒	☐	Animals and other organisms
☐	☐	Clinical data
☒	☐	Dual use research of concern

Methods

n/a	Involved in the study	
☒	☐	ChIP-seq
☒	☐	Flow cytometry
☒	☐	MRI-based neuroimaging

Clinical data

Policy information about clinical studies

All manuscripts should comply with the ICMJE guidelines for publication of clinical research and a completed CONSORT checklist must be included with all submissions.

Clinical trial registration	We did not undertake a clinical trial and therefore did not register on clinicaltrials.gov. However, our systematic review protocol was registered o the Prospero database

Study protocol	https://www.crd.york.ac.uk/prospero/display_record.php?RecordID=310539
Data collection	We extracted full-text data over 3 months
Outcomes	Outcomes were specified during the design of our systematic review protocol, published on prospero.
